# Supplementary material for: Fetal Exposure to Air Pollution in Late Pregnancy Significantly Increases ADHD-Risk Behavior in Early Childhood
Source: Int J Environ Res Public Health. 2022 Aug 23;19(17):10482. doi: 10.3390/ijerph191710482 (PMC9518584; doi:10.3390/ijerph191710482)
Supplement: Supplementary file 1 [file ijerph-19-10482-s001.zip › ijerph-1806270-supplementary.pdf]

Part S1 Air pollution exposure assessment

1. Materials and methods

1.1 Study area

Considering the small number and unbalanced spatial distribution of air monitoring stations in Shenzhen, the Pearl River Delta (PRD) was selected as study area in order to ensure the prediction simulation accuracy of LURF model. The PRD area (21°31′ - 23°10′N, 112°45′ - 113°50′E) is located in Guangdong Province, North China, with an area of 5.5×10^5 km^2, constituting 30.81% of the total area of Guangdong Province. The population of the study area in 2018 was 63 million, and the urbanization rate was 84.9%. The heating season in the study area generally starts in November and ends in March of the next year. The nine study cities in the PRD are Guangzhou, Shenzhen, Dongguan, Foshan, Zhongshan, Zhuhai, Zhaoqing and Zhuhai (Fig.S1).

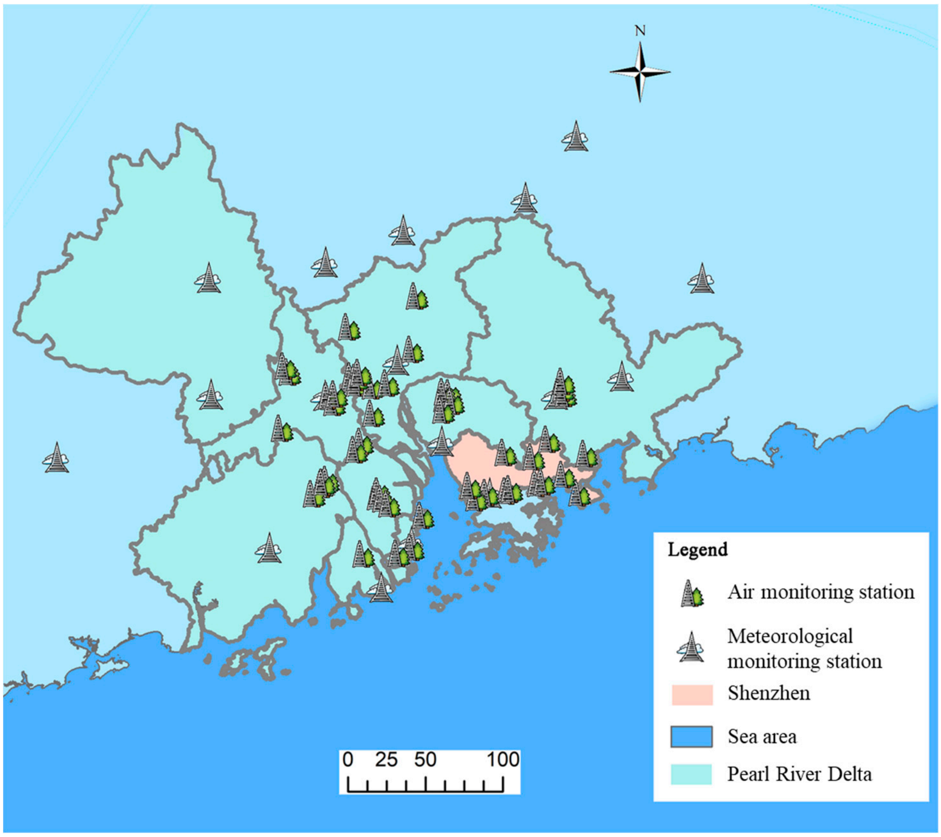

Fig S1. Study Area

1.2 Air pollutant data

China began to carry out long-term monitoring of $PM_{2.5}$, CO and O_3 concentrations in 2013. Data on six pollutant (SO_2, NO_2, CO, O_3, $PM_{2.5}$, PM_{10}) concentrations in nine cities for 2013~2019 were collected from 57 monitoring stations in the China National Environment Monitoring Centre (http://www.cnemc.cn/) (Fig.S1). Among them, there are 11 each monitoring stations in Guangzhou and Shenzhen, 8 in Foshan, 5 each in Dongguan, Huizhou and Zhaoqing and 4 each in Zhuhai, Zhongshan and Jiangmen. Invalid data were eliminated based on the standards for monitored pollutant concentration data in the Ambient Air Quality Standards (GB 3095-2012)[1]. The monthly means of 6 air pollutant concentrations at the monitoring stations were used as dependent variables in the following LURF modelling. The concentrations of 2011-2012 was backtracked by deep learning multi-output LSTM(DM-LSTM) model.

1.3 Predictor variables

Taking into account direct and potential influencing factors on air pollutant emissions and diffusion and distribution in urban areas, six categories including seventeen parameters were selected for our study[2-4]: (1) land use, (2) traffic information, (3) population, (4) local pollutant sources, (5) geographic information and (6) meteorological data. Six parameters using buffer sizes and eleven parameters using distance, geographical coordinates or urban lot property data were used to fit an LURF model for $PM_{2.5}$ in the PRD (Table S1). All variables are numerical types. Six types of land use data were obtained by interpreting Landsat 8 images. According to a field survey and comparison with other data from the same year, the overall accuracy of classification was 71%. The proportion of area of each land use type in the buffer was counted as a predictor. Traffic data were obtained from OpenStreetMap (https:// www.openstreetmap.org). Population density data were extracted from the Statistical Yearbook of Guangdong Province. Pollutant emission sources were identified from the Information disclosure Platform of Guangdong Provincial Department of Ecology and Environment (http://gdee.gd.gov.cn/jcsj). Elevation data were obtained from a 250 m DEM from the Data Center for Resources and Environmental Sciences, Chinese

Academy of Sciences (RESDC) (https://www.resdc.cn). Meteorological data were obtained from National Meteorological Data Center of China(http://data.cma.cn/).

1.4 Land use random forest modelling and evaluation

To derive residential exposure to air pollution (SO_2, NO_2, CO, O_3, $PM_{2.5}$, PM_{10}), we used a land use random forest (LURF) model developed by Shin Araki and colleagues[5]. In short, A five-step backward method was adopted for fitting the LURF model: (1) A simple linear regression model was used to evaluate the relationship between each predictor and each air pollutant concentrations; (2) the predictor variables were sorted by Pearson correlation coefficient, and the highest ranked variables in each subcategory were included; (3) other variables (Pearson correlation coefficient $R \geq 0.7$) correlated with the highest ranked variables in each subcategory were removed; (4) all the remaining predictor variables were entered into the random forest model; and (5) the significance level (p-value<10%) and variance inflation factor (VIF<4) of each predictor variable were checked to confirm the variables' significance levels and ensure no issues of multicollinearity.

The leave-one cross-validation (LOOCV) was chosen to evaluate the predictive capacity of the model. From the cross validation, the mean absolute percentage error (MAPE) and root-mean-squared error (RMSE) were used to evaluate and compare the predictivity of the model[6].

1.5 Pollution surface mapping

Since most of the children' birth address located in Shenzhen, we selected Shenzhen city areas to generate the predicted six air pollution surface. Prediction points were generated on a 100 m grid within the study boundaries. The land use parameters included in the regression kriging model were calculated for each grid cell center point. Using the land use data, air pollution concentrations were estimated for each point of the 100-m grid.

Table S1. Description of the predictor variables in the model

No.	Predictor variables	Abbreviations	Unit	Buffer size
Land use				
1	Corpland	Cor	m^3	500,1000,1500,2000,3000
2	Forest	For		
3	Grassland	Gra		
4	Water	Wat		
5	Unused Land	Unu		
6	Construction land	Con		
Traffic informaion				
7	Road length	Roa	km	NA
Population				
8	Population density	Pop	count/km^2	NA
Local pollutant source				
9	Distance to pollutant emission source	D_Pollutant	km	NA
Geographic information				
10	Elevation	DEM	m	NA
11	Distance to coastline	D_Coastline	km	NA
Meteorological data				
12	Wind Speed	Win	m/s	NA
13	Max wind Speed	Max_Win	m/s	NA
14	Rainfall	Rain	mm	NA
15	Sunshine duration	Sun	h	NA
16	Humidity	Hum	%	NA
17	Temperature	Tem	℃	NA

2. Results

2.1 LURF model

Scatter plots of the predicted and observed concentrations obtained through cross validation are presented in Fig.S3. The dot color indicates the point density in the plot: red and blue indicate higher and lower density, respectively. MAPE and RMSE values are given in each panel for all stations.

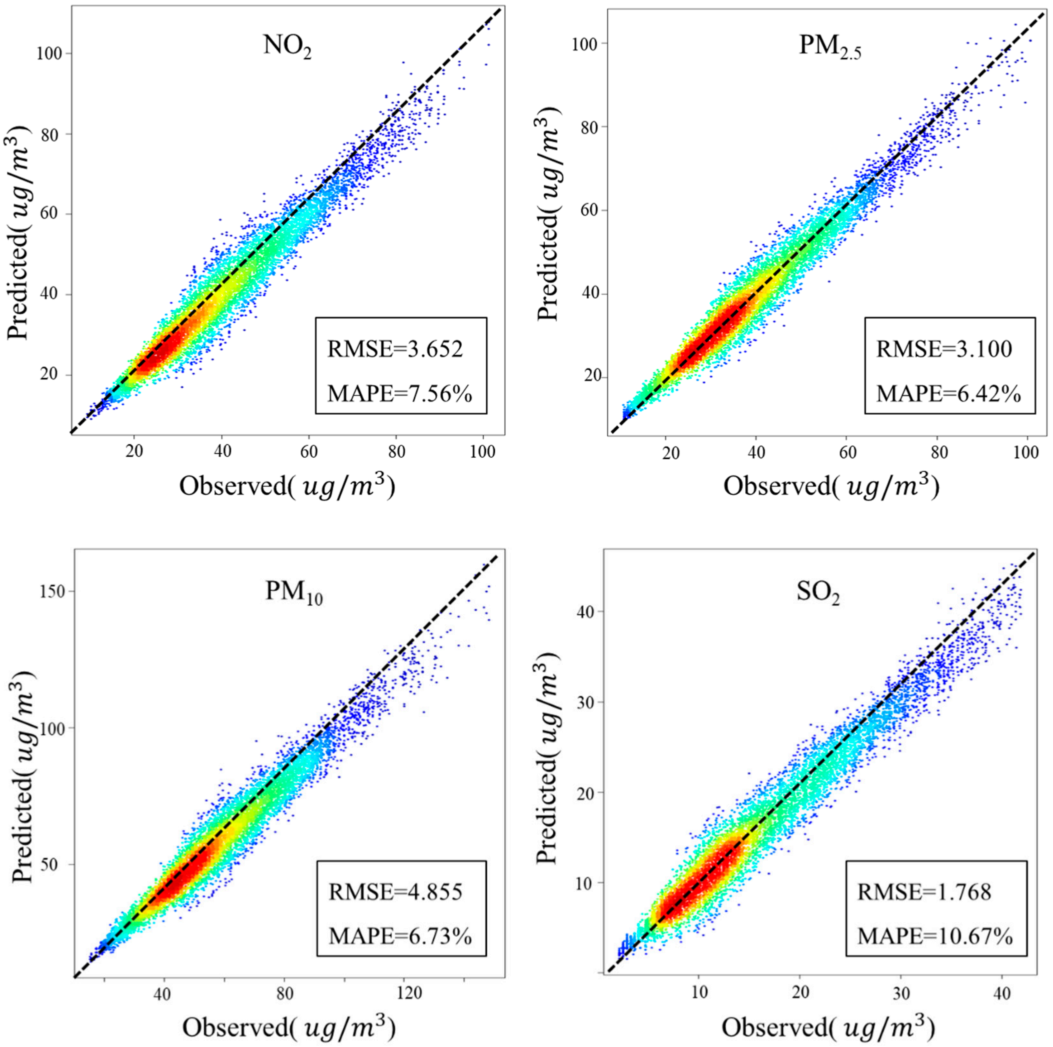

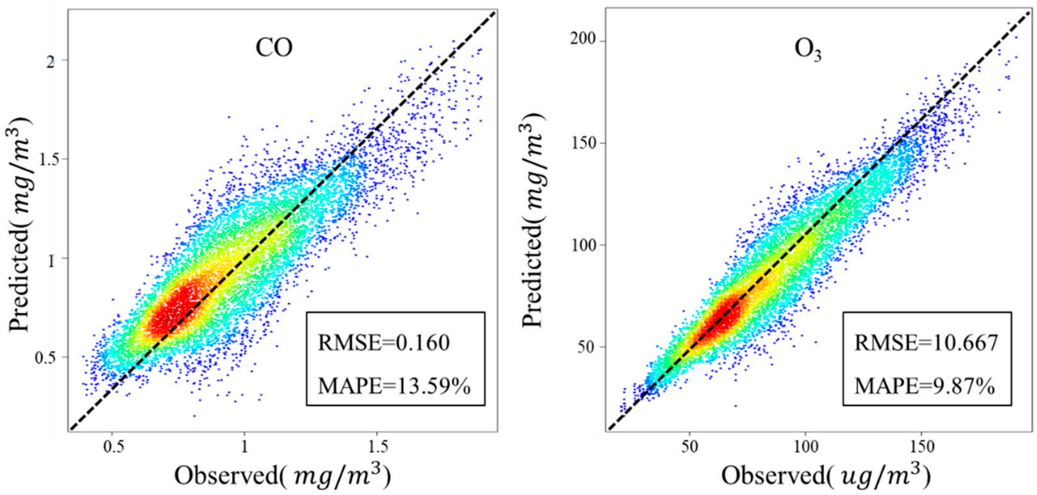

Fig S2. Scatter plots of predicted and observed concentrations obtained from cross validation

2.2 Mapping

Fig.S3 is a prediction map of the six air pollutant concentrations averaged in March 2013 as an example, considering our study contains 504 LURF models (6 air pollutant×12months×7years).

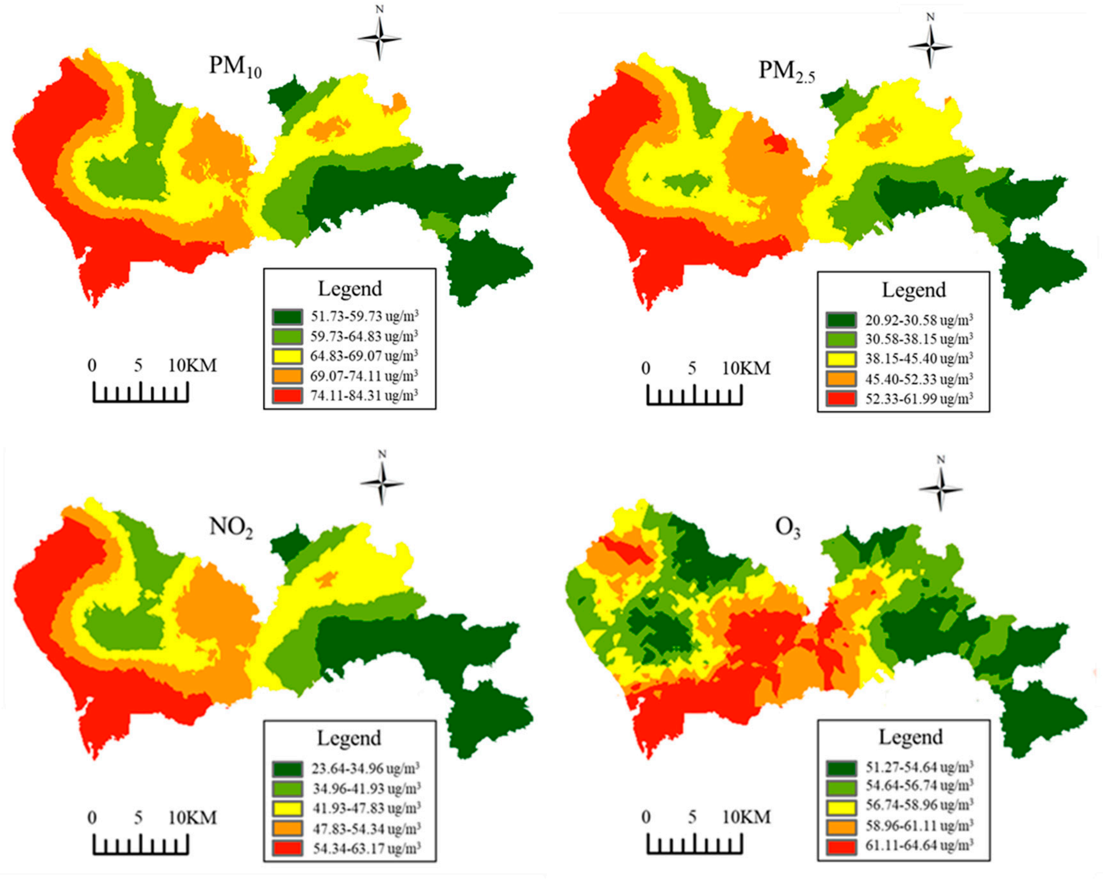

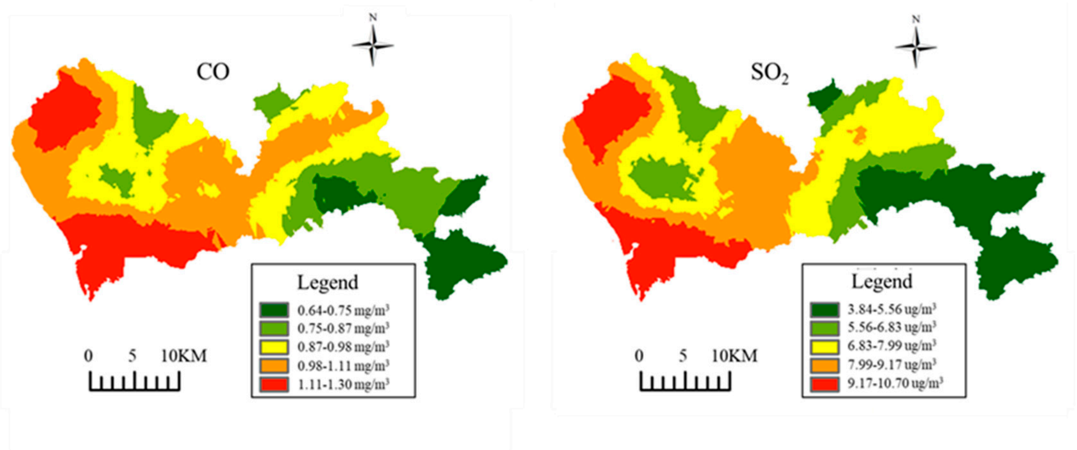

Fig S3. Six air pollution surfaces of Shenzhen generated using regression kriging (spatial resolution is 100 m)

Part S2 Sensitivity analysis of DLNM model

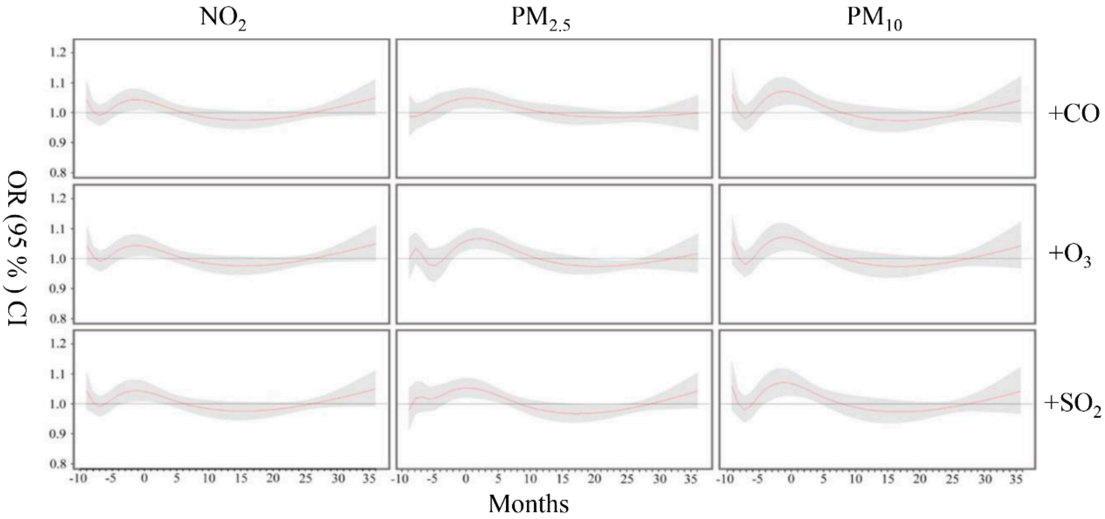

Figure S4. Two-pollutants model analyses on the odds ratio of child hyperactivity associated association with monthly $PM_{2.5}$, PM_{10} and NO_2 exposure from fetal period to the first 3 years of life.

ORs (95%CI) in the three uppermost charts indicate the risks of PM2.5, PM10 and NO2 for each 10μg/m3 increment in monthly PM2.5, PM10 and NO2 concentrations during pregnancy.

Zero in the X-axis scale indicates the time of birth, minus numbers indicate the weeks prior to birth, and plus numbers indicate the postnatal months.

All models were adjusted for monthly mean ambient temperature, household air pollution conditions, child sex and age, maternal and paternal age at birth, maternal and paternal education, family income, marital status, parity, multivitamins supplementation during pregnancy, gestational diseases, passive smoking during pregnancy, gestation alcohol consumption, feeding pattern, average daily sleep duration for children and frequencies of parent-child interactive activities at 0-1 and 1-3 years.

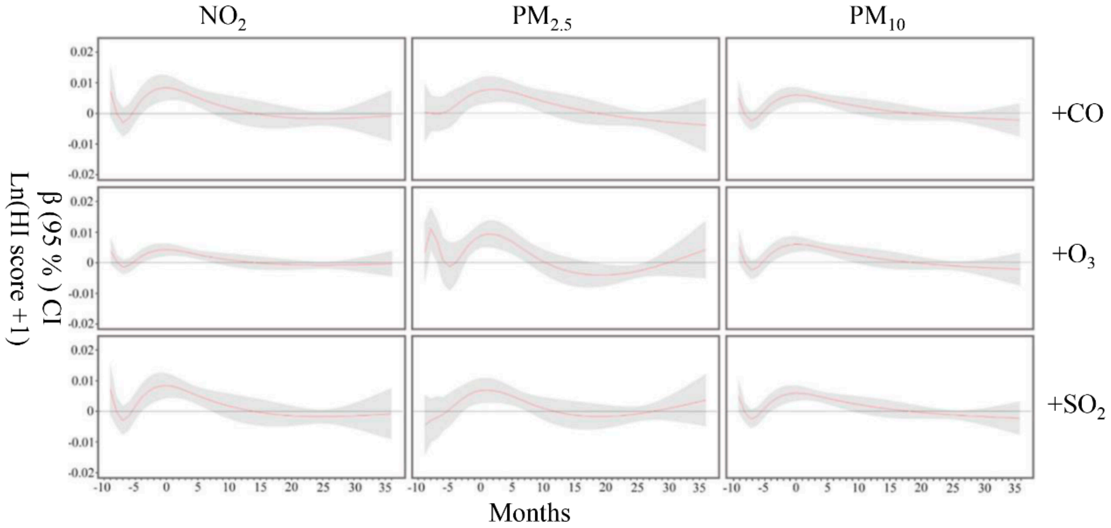

Figure S5. Two-pollutants model analyses on the changes in child ln (HI score +1) around 3 years old associated association with monthly $PM_{2.5}$, PM_{10} and NO_2 exposure from fetal period to the first 3 years of life.

β (95%CI) in the three uppermost charts indicate the risks of PM2.5, PM10 and NO2 for each 10μg/m3 increment in monthly PM2.5, PM10 and NO2 concentrations during pregnancy.

Zero in the X-axis scale indicates the time of birth, minus numbers indicate the weeks prior to birth, and plus numbers indicate the postnatal months.

All models were adjusted for monthly mean ambient temperature, household air pollution conditions, child sex and age, maternal and paternal age at birth, maternal and paternal education, family income, marital status, parity, multivitamins supplementation during pregnancy, gestational diseases, passive smoking during pregnancy, gestation alcohol consumption, feeding pattern, average daily sleep duration for children and frequencies of parent-child interactive activities at 0-1 and 1-3 years.

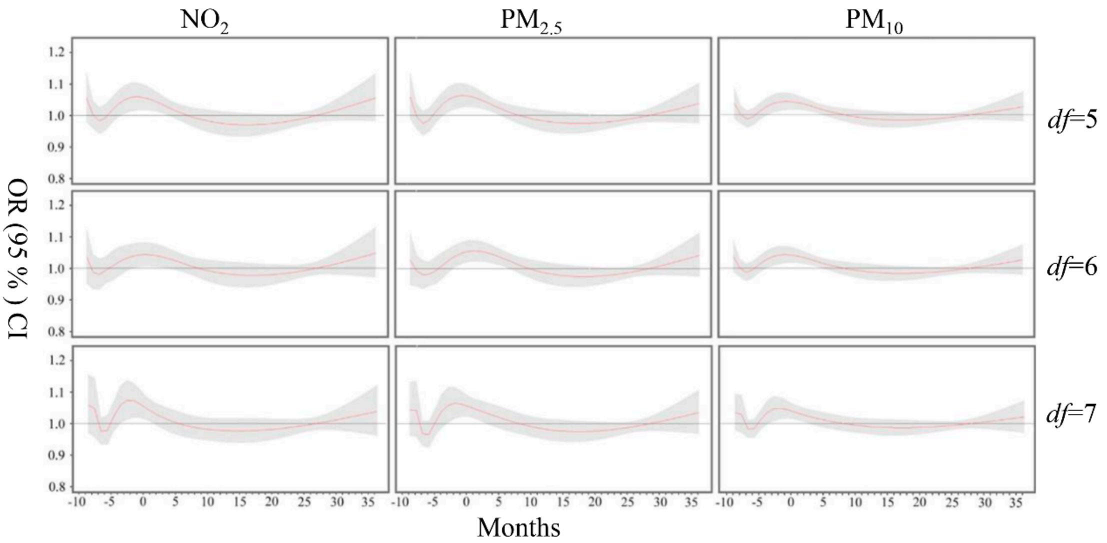

Figure S6. The odds ratio of child hyperactivity associated association with monthly $PM_{2.5}$, PM_{10} and NO_2 exposure from fetal period to the first 3 years of life with different degree of freedom.

ORs (95%CI) in the three uppermost charts indicate the risks of PM2.5, PM10 and NO2 for each 10μg/m3 increment in monthly PM2.5, PM10 and NO2 concentrations during pregnancy.

Zero in the X-axis scale indicates the time of birth, minus numbers indicate the weeks prior to birth, and plus numbers indicate the postnatal months.

All models were adjusted for monthly mean ambient temperature, household air pollution conditions, child sex and age, maternal and paternal age at birth, maternal and paternal education, family income, marital status, parity, multivitamins supplementation during pregnancy, gestational diseases, passive smoking during pregnancy, gestation alcohol consumption, feeding pattern, average daily sleep duration for children and frequencies of parent-child interactive activities at 0-1 and 1-3 years.

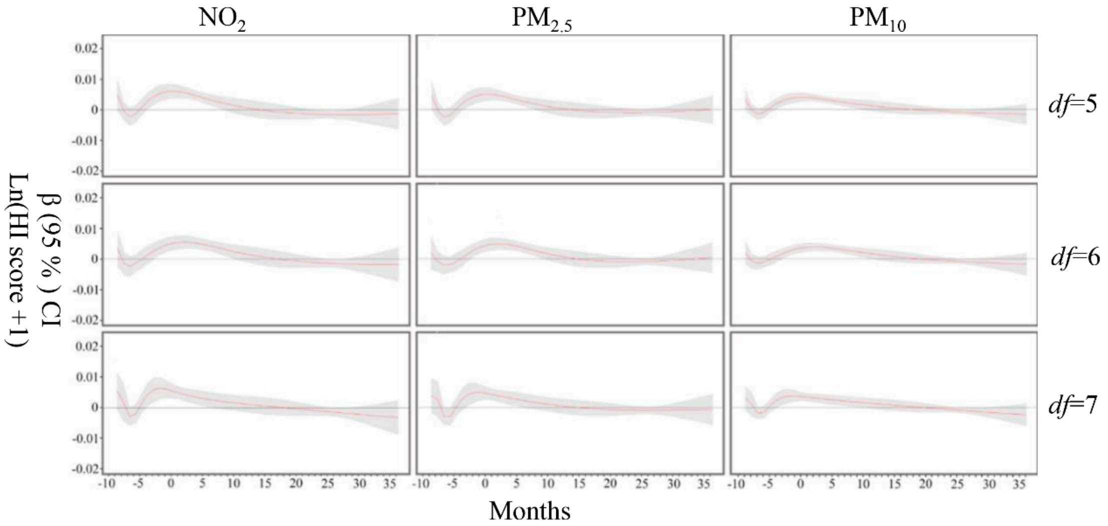

Figure S7. The changes in child ln (HI score +1) around 3 years old associated association with monthly $PM_{2.5}$, PM_{10} and NO_2 exposure from fetal period to the first 3 years of life with different degree of freedom.

β (95%CI) in the three uppermost charts indicate the risks of PM2.5, PM10 and NO2 for each 10μg/m3 increment in monthly PM2.5, PM10 and NO2 concentrations during pregnancy.

Zero in the X-axis scale indicates the time of birth, minus numbers indicate the weeks prior to birth, and plus numbers indicate the postnatal months.

All models were adjusted for monthly mean ambient temperature, household air pollution conditions, child sex and age, maternal and paternal age at birth, maternal and paternal education, family income, marital status, parity, multivitamins supplementation during pregnancy, gestational diseases, passive smoking during pregnancy, gestation alcohol consumption, feeding pattern, average daily sleep duration for children and frequencies of parent-child interactive activities at 0-1 and 1-3 years.

References

[1] China, M. E. P. Ambient Air Quality Standards. GB 3095-2012. *China Environmental Science Press*, *Beijing* **2012**.(In Chinese) Available online: https://www.mee.gov.cn/ywgz/fgbz/bz/bzwb/dqhjbh/dqhjzlbz/201203/W020120410330232398521.pdf.

[2] Chen, L., Bai, Z., Kong, S., Han, B., You, Y., Ding, X., et al., 2010. A land use regression for predicting NO2 and PM10 concentrations in different seasons in Tianjin region, China. J. Environ. Sci. 22, 1364–1373, doi: 10.1016/S1001-0742(09)60263-1.

[3] Liu, C.; Henderson, B.H.; Wang, D.F.; Yang, X.Y.; Peng, Z.R. A land use regression application into assessing spatial variation of intra-urban fine particulate matter (PM2.5) and nitrogen dioxide (NO2) concentrations in City of Shanghai, China. *Sci Total Environ* **2016**, 565, 607–615, doi: 10.1016/j.scitotenv.2016.03.189.

[4] Shi, Y.; Lau, K. L.; Ng, E. Incorporating wind availability into land use regression modelling of air quality in mountainous high density urban environment. *Environ Res* **2017**, 157, 17–29,doi: 10.1016/j.envres.2017.05.007.

[5] Araki, S.; Shima, M.; Yamamoto, K. Spatiotemporal land use random forest model for estimating metropolitan NO2 exposure in Japan. *The ence of the Total Environment* **2018**, 634(SEP.1), 1269-1277.
[6] Hoek, G.; Beelen, R.; de Hoogh, K.; Vienneau, D.; Gulliver, J.; Fischer, P., Briggs, D. A review of land-use regression models to assess spatial variation of outdoor air pollution. *Atmos Environ* **2008**, 42, 7561–7578, doi: 10.1016/j.atmosenv.2008.05.057.
